# Long Term Outcomes of Lung Transplantation in Sensitized Patients Following Eculizumab Use With the Desensitization Protocol

**DOI:** 10.3389/ti.2025.15040

**Published:** 2025-09-22

**Authors:** Yudai Miyashita, Taisuke Kaiho, David F. Pinelli, Anthony Joudi, Mihir John, Austin Chang, Benjamin Louis Thomae, Amanda Kamar, Carl Atkinson, Ankit Bharat, G. R. Scott Budinger, Ambalavanan Arunachalam, Chitaru Kurihara

**Affiliations:** ^1^ Division of Thoracic Surgery, Department of Surgery, Northwestern University Feinberg School of Medicine, Chicago, IL, United States; ^2^ Division of Transplant, Department of Surgery, Northwestern University Feinberg School of Medicine, Chicago, IL, United States; ^3^ Division of Pulmonary and Critical Care Medicine, Department of Medicine, Northwestern University Feinberg School of Medicine, Chicago, IL, United States

**Keywords:** lung transplantation, sensitized, eculizumab, ACR, AMR

## Abstract

Lung transplantation remains a life-saving option for end-stage pulmonary diseases, but sensitized patients with anti HLA antibodies carry high risk; recent desensitization advances, such as eculizumab, may permit outcomes comparable to non-sensitized recipients with tailored perioperative care. In this prospective cohort study of 399 adult lung transplant recipients, 36 sensitized patients underwent a protocol combining preoperative plasmapheresis, a defined eculizumab regimen, anti-thymocyte globulin, and IVIG. In comparison, 363 non-sensitized recipients received standard immunosuppression. We compared recipient/donor characteristics, intraoperative parameters, and postoperative outcomes, including primary graft dysfunction, infection, rejection, and overall survival. Desensitized patients were older, predominantly female, and had significantly higher panel reactive antibody levels and preformed donor-specific antibodies; intraoperatively, they required more blood transfusions and VA-ECMO support. Postoperatively, they exhibited higher rates of *de novo* donor-specific antibodies, antibody-mediated rejection, longer ICU stays, increased dialysis requirement, and more frequent CMV infections. Despite these differences, rates of acute cellular rejection, chronic lung allograft dysfunction, and one-year and overall survival were similar between groups. Our findings suggest that lung transplantation in sensitized patients managed with a desensitization protocol, including eculizumab, is feasible and safe, achieving outcomes comparable to those of non-sensitized recipients.

## Graphical Abstract

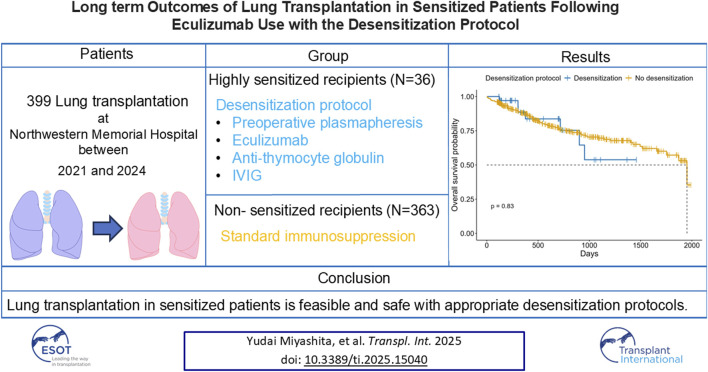

## Introduction

Lung transplantation is a life-saving procedure for patients with end-stage pulmonary diseases, offering improved survival and quality of life [1]. However, some transplant candidates are sensitized, harboring elevated levels of pre-formed anti-HLA antibodies. Historically, these patients have been considered at higher risk for complications such as hyperacute rejection and severe infections, rendering them less favorable candidates for transplantation [2, 3]. Recent advances in immunology and desensitization protocols have prompted a re-evaluation of lung transplantation in sensitized patients [4, 5]. While traditional approaches have often excluded these patients from transplant candidacy, emerging evidence suggests that perioperative desensitization strategies—such as repeated plasmapheresis, administration of high-dose IVIG, and targeted immunomodulatory agents—can mitigate the risks associated with pre-formed donor-specific antibodies (DSA) [6, 7]. Despite the theoretical risk of higher rates of rejection and infection, these interventions hold the potential to enable safe transplantation in a group previously deemed ineligible. Nevertheless, data on the clinical outcomes of lung transplantation in sensitized recipients remain limited, particularly regarding long-term survival and complication rates. While study have reported comparable survival and CLAD-free survival between sensitized and non-sensitized recipients following desensitization protocols, detailed evaluations of complications in this context are still lacking [8]. Several prior single-center experiences have demonstrated the feasibility of perioperative desensitization in lung-transplant cohorts. In 2015, Tinckam et al. described 340 first-time transplants—including 53 DSA-positive patients—managed with perioperative plasma exchange (PLEX), IVIG, antithymocyte globulin (ATG), and mycophenolate, reporting similar one-year graft survival and freedom from acute rejection compared with unsensitized controls [9]. Aversa et al. subsequently evaluated 74 virtual-crossmatch-positive/flow-crossmatch-positive recipients treated with PLEX, IVIG, and ATG and found 5-year allograft and CLAD-free survival equivalent to VXM-negative patients [10]. Parquin et al. implemented a virtual-crossmatch–based protocol in 39 high-DSA candidates at Foch Hospital—using PLEX, rituximab, and IVIG—and demonstrated comparable 3-year graft survival and CLAD-free survival versus non-sensitized recipients [11]. More recently, Heise et al. reported a 9-year, single-center experience in 62 sensitized recipients treated with IgA- and IgM-enriched IVIG (IgGAM), PLEX, and a single dose of rituximab, achieving 73% DSA clearance and long-term outcomes analogous to those of unsensitized patients [12]. Together, these studies showed the diverse of perioperative regimens—incorporating PLEX, IVIG (or IgGAM), ATG (or basiliximab), and rituximab,—can safely expand transplant access for sensitized candidates without compromising medium-term outcomes. Despite these encouraging results, Marfo et al. indicates an increased incidence of infections and episodes of antibody-mediated rejection (AMR) [13]. Still, overall survival may remain comparable to that of non-sensitized recipients if rigorous surveillance and specialized immunosuppressive regimens are in place. Recent evidence has highlighted the potential of preventative treatment with eculizumab, a terminal complement inhibitor, in mitigating the risk of AMR in sensitized patients undergoing heart and kidney transplantation [14, 15]. Building on this, we previously demonstrated the feasibility of performing successful multiorgan transplantation in sensitized patients with positive crossmatch donors by implementing a perioperative desensitization protocol incorporating eculizumab [16]. This approach not only mitigated the heightened immunological risk but also highlighted the importance of tailored strategies in expanding transplant opportunities for this challenging patient population while maintaining acceptable long-term outcomes.

In this study, we evaluated our institution’s experience with sensitized patients who underwent lung transplantation following a desensitization protocol with Eculizumab. We compared perioperative and postoperative outcomes—including rates of primary graft dysfunction (PGD), infection, rejection, and survival—between sensitized patients receiving desensitization therapy and non-sensitized patients. We aimed to determine whether lung transplantation can be performed safely and effectively in the sensitized population without compromising overall postoperative outcomes.

## Patients and Methods

### Study Design

This is a cohort study of adult patients who underwent lung transplantation at a single institution between September 2021 and August 2024. Patient data were collected prospectively using electronic medical records. The study was approved by the Institutional Review Board of Northwestern University (STU00207250, STU00213616, and STU00217958). The need for patient consent for data collection was waived by the institutional review board due to the retrospective nature of this study. Recipient and donor characteristics, preoperative laboratory values, and intra- and postoperative outcomes were compared in lung transplant patients.

### Peri- and Post-Operative Protocol for Sensitized Patients

The protocol has been previously reported by our group [16]. Specifically, sensitized patients with PRA above 40 (details in supplemental methods about HLA testing) received plasmapheresis 4–6 h prior to lung transplant. Both sensitized and non-sensitized patients received steroid and simlect as induction therapy at the time of lung transplant. Sensitized patients received total 5 sessions of plasma exchange (Pre-operative, POD0, 1, 2, and 3), eculizumab (Pre-operative: 1200mg, POD 0: 900 mg, POD 1: 600 mg, 2: 600 mg, 3: 1200 mg), anti-thymocyte globulin (POD5-, 1 mg/kg/day, total cumulative dose 4–8 mg/kg), IVIG 300 mg/kg if plasma IgG <500 (Supplementary Table S1; Supplementary Figure S1).

### Statistical Analysis

Continuous data are shown as median (Interquartile Range; IQR), and discrete data are shown as number (%). Recipient and donor characteristics, preoperative laboratory values, and intra- and postoperative outcomes were compared between lung transplant patients. The Mann-Whitney U test was used to compare independent continuous variables between the groups. Fisher’s exact test was used to compare categorical variables, which were reported as numbers and percentages. The Kaplan-Meier method was used to estimate survival, and the log-rank test was performed to compare survival between the groups. Hazard ratio (HR) was obtained using a univariate and multivariate cox proportional hazard analysis and odds ratio was obtained using a univariate and multivariate logistic regression analysis. Statistical significance was set at p < 0.05. All statistical analyses were performed using the JMP Pro 17.0.0 software program (SAS Institute Inc.).

## Result

### Patient Characteristics

399 lung transplant recipients were analyzed, comprising 36 patients who underwent desensitization protocols and 363 who did not (Table 1). The median age of the desensitization group was significantly higher than the non-desensitization group [63.0 years (56.0–68.0) vs. 53.5 years (48.3–67.8), p = 0.023]. The proportion of female recipients was significantly greater in the desensitization group (86.1% vs. 38.3%, p < 0.0001). Patients in the desensitization group had significantly higher Panel Reactive Antibody (PRA) levels for both Class I and Class II [Class I: 44.0% (23.3–87.0) vs. 0.0%, p < 0.0001; Class II: 10.5% (0.0–83.8) vs. 0.0%, p < 0.0001]. All patients in the desensitization group tested positive for PRA (100.0% vs. 32.8%, p < 0.0001), and a significantly higher proportion had positive T cell flow cytometry crossmatch (FC-XM) (63.9% vs. 0.0%, p < 0.0001), B cell FC-XM (72.2% vs. 0.0%, p < 0.0001), and both T and B cell FC-XM (61.1% vs. 0.0%, p < 0.0001). Preformed DSA were also markedly more frequent in the desensitization group (75.0% vs. 6.4%, p < 0.0001) (Supplementary Table S2). Regarding etiology, interstitial lung disease (ILD) was less prevalent in the desensitization group (19.4% vs. 39.9%), whereas COVID-19-related indications were more common (27.8% vs. 10.2%, p = 0.0023).

**TABLE 1:** Characteristics of patients.

Variable	Desensitization protocol (n = 36)	No Desensitization protocol (n = 363)	p value
Recipient factors			
Age, years	63.0 (56.0–68.0)	53.5 (48.3–67.8)	0.023
Female	31 (86.1%)	139 (38.3%)	<0.0001
BMI, kg/m2*	27.8 (24.3–29.8)	26.4 (22.1–29.4)	0.13
BSA, m2*	1.8 (1.6–19)	1.9 (1.7–2.1)	0.0079
Smoking history	13 (36.1%)	185 (51.0%)	0.12
Hypertension	18 (50.0%)	204 (56.2%)	0.49
Diabetes	10 (27.8%)	112 (30.9%)	0.85
CKD	1 (2.8%)	32 (8.8%)	0.34
Bilateral	26 (72.2%)	222 (61.2%)	0.21
PRA			
Class I	44.0 (23.3–87.0)	0.0 (0.0–0.0)	<0.0001
Class II	10.5 (0.0–83.8)	0.0 (0.0–0.0)	<0.0001
any PRA	36 (100.0%)	119 (32.8%)	<0.0001
Positive T cell FC-XM	23 (63.9%)	0 (0.0%)	<0.0001
Positive B cell FC-XM	26 (72.2%)	0 (0.0%)	<0.0001
Positive T and B cell FC-XM	22 (61.1%)	0 (0.0%)	<0.0001
Any positive T and B cell FC-XM	27 (75.0%)	0 (0.0%)	<0.0001
preformed DSA	27 (75.0%)	23 (6.4%)	<0.0001
Etiology			0.0023
ILD	7 (19.4%)	145 (39.9%)	
COPD	3 (8.3%)	72 (19.8%)	
PAH	3 (8.3%)	22 (6.1%)	
COVID-19	10 (27.8%)	37 (10.2%)	
other	13 (36.1%)	87 (24.0%)	
Laboratory			
Hemoglobin, g/dL*	10.5 (8.8–13.1)	11.9 (9.9–13.4)	0.053
WBC, 1,000/mm3*	9.8 (7.8–12.6)	8.7 (7.0–11.3)	0.25
Platelets, 1,000/mm3*	256.0 (199.0–304.8)	238.5 (189.0–302.8)	0.57
Sodium, mEq/L	140.0 (138.0–141.8)	139.0 (138.0–141.0)	0.42
BUN, mg/dL	14.0 (12.0–17.8)	16.0 (13.0–20.0)	0.079
Creatinine, mg/dL	0.7 (0.6–0.8)	0.8 (0.6–0.9)	0.0045
ALT, U/L*	16.0 (12.0–21.0)	17.0 (11.0–25.0)	0.90
AST, U/L*	19.5 (16.3–34.8)	21.0 (17.0–28.0)	0.90
Albumin, g/dL*	4.1 (3.7–4.3)	4.0 (3.6–4.3)	0.16
Total bilirubin, mg/dL	0.4 (0.4–0.8)	0.5 (0.3–0.7)	0.84
INR	1.1 (1.0–1.1)	1.0 (1.0–1.1)	0.62
Arterial blood gas			
pH	7.4 (7.4–7.4)	7.4 (7.3–7.4)	0.39
PaCO2	50.5 (40.5–58)	48 (42.0–55.0)	0.52
PaO2	307.0 (193.3–375.5)	281.0 (195.0–358.0)	0.50
Donor			
Age, years	37.0 (31.3–46.8)	33.0 (24.0–45.0)	0.17
Female	16 (44.4%)	111 (30.6%)	0.094
Cause of death			0.29
Anoxia	19 (52.8%)	146 (40.2%)	
Head trauma	9 (25.0%)	128 (35.3%)	
Stroke	6 (16.7%)	78 (21.5%)	
Other	2 (5.6%)	11 (3.0%)	

Continuous data are shown as median (interquartile range) and discrete data are shown as number (%). BMI, body mass index; BSA, body surface area; CKD, chronic kidney disease; PRA, panel reactive antibody; FC-XM, flow cytometry crossmatching; DSA, donor specific antibody; ILD; interstitial lung disease; COPD, chronic obstructive pulmonary disease; PAH, pulmonary arterial hypertension; WBC, white blood cell; BUN, blood urea nitrogen; AST, aspartate aminotransferase; ALT, alanine aminotransferase; INR, international normalized ratio. *Unknown cases were excluded.

### Intraoperative and Postoperative Outcomes

Intraoperative outcomes are shown in Table 2. Patients undergoing desensitization required significantly more intraoperative blood transfusion, including packed red blood cells (pRBC) [0.0 units (0.0–4.5) vs. 0.0 units (0.0–2.0), p = 0.0075] and fresh frozen plasma (FFP) [0.0 units (0.0–3.8) vs. 0.0 units (0.0–0.0), p = 0.01]. Ischemic time was comparable [5.4 h (4.4–6.2) vs. 5.2 h (4.1–6.1), p = 0.60]. VA-ECMO was used significantly more often in the desensitization group (80.6% vs. 60.6%, p = 0.019).

**TABLE 2:** Intraoperative and Postoperative outcomes.

Variable	Desensitization protocol (n = 36)	No Desensitization protocol (n = 363)	p value
Intraoperative outcomes			
Operative time (hours)	6.3 (4.8–7.5)	5.7 (4.4–7.5)	0.40
Intra-op blood transfusion (unit)			
pRBC	0.0 (0.0–4.5)	0.0 (0.0–2.0)	0.0075
FFP	0.0 (0.0–3.8)	0.0 (0.0–0.0)	0.01
Plt	0.0 (0.0–0.0)	0.0 (0.0–0.0)	0.47
Ischemic time (hours)	5.4 (4.4–6.2)	5.2 (4.1–6.1)	0.60
VA-ECMO use	29 (80.6%)	220 (60.6%)	0.019
VA-ECMO time (hours)	2.7 (2.3–3.3)	2.9 (0.4–3.5)	0.71
Postoperative outcomes			
*de novo* DSA	20 (55.6%)	45 (12.4%)	<0.0001
PGD			
Any grade	26 (72.2%)	196 (54.0%)	0.053
Grade>=2	14 (38.9%)	98 (27.0%)	0.17
Grade3	4 (11.1%)	46 (12.7%)	1.00
AKI	17 (47.2%)	170 (46.8%)	1.00
Dialysis	11 (30.6%)	49 (13.5%)	0.012
CVA	0 (0.0%)	12 (3.3%)	0.61
Bowel ischemia	1 (2.8%)	5 (1.4%)	0.44
Digital ischemia	1 (2.8%)	5 (1.4%)	0.44
ICU stay (days)	10.0 (5.3–25.8)	7.0 (4.8–15.0)	0.050
Post transplant ventilator (days)	2.5 (2.0–3.8)	2.0 (1.0–3.0)	0.049
Hospital stay (days)	20.0 (13.0–44.0)	17.0 (12.0–31.0)	0.087
1-year survival	91.7%	89.0%	0.78
Follow-up period (days)	367.5 (228.5–729.8)	567.0 (235.0–1077.0)	0.061

Continuous data are shown as median (interquartile range) and discrete data are shown as number (%). pRBC, packed red blood cells; FFP, fresh frozen plasma; Plt, platelets; VA ECMO, veno-arterial extracorporeal membrane oxygenation; DSA, donor specific antibody; PGD, primary graft dysfunction; AKI, acute kidney injury; CVA, cerebrovascular attack; ICU, intensive care unit.

Postoperative outcomes revealed that *de novo* DSA was significantly more frequent in the desensitization group (55.6% vs. 12.4%, p < 0.0001). PGD grade at 72 h post-transplantation showed a trend toward higher grades in the desensitization group, though this was not statistically significant (p = 0.079). Specifically, PGD grade 0 was less frequent in the desensitization group (27.8% vs. 46.0%), while grades 1 and 2 were more common. AKI occurred at a similar rate in both groups (47.2% vs. 46.8%, p = 1.00); however, the need for dialysis was significantly higher in the desensitization group (30.6% vs. 13.5%, p = 0.012). Patients in the desensitization group had a longer median ICU stay [10.0 days (5.3–25.8) vs. 7.0 days (4.8–15.0), p = 0.050] and required longer post-transplant ventilator support [2.5 days (2.0–3.8) vs. 2.0 days (1.0–3.0), p = 0.049]. Hospital stay also tended to be longer in the desensitization group [20.0 days (13.0–44.0) vs. 17.0 days (12.0–31.0), p = 0.087], although this difference did not reach statistical significance. Despite these differences, the two groups’ one-year survival rates were comparable (91.7% vs. 89.0%, p = 0.78).

### Infection Outcomes

**FIGURE 1:**
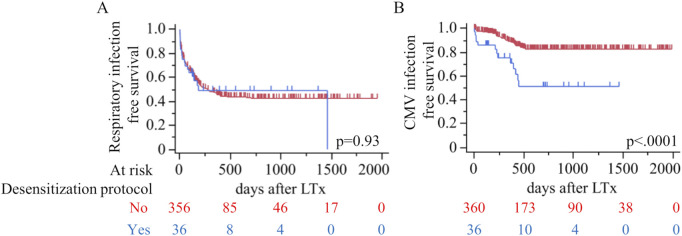
Kaplan–Meier curves comparing outcomes after lung transplantation by use of a perioperative desensitization protocol (red, No; blue, Yes). **(A)** Respiratory infection–free survival (log-rank p=0.93). **(B)** Cytomegalovirus (CMV) infection–free survival (p<0.0001). LTx, lung transplantation.

The infection outcome is shown in Table 3 and Figures 1A,B. The overall incidence of infections was similar between the desensitization and non-desensitization groups (69.4% vs. 61.9%, p = 0.47). Respiratory infections occurred at similar rates between the two groups (50.0% vs. 52.2%, p = 0.86), as did recurrent respiratory infections (19.4% vs. 16.4%, p = 0.64). Figure 1A demonstrates that respiratory infection-free survival did not differ significantly between the two groups (p = 0.93). However, CMV infections were significantly more frequent in the desensitization group compared to the non-desensitization group (36.1% vs. 13.1%, p = 0.0009). Baseline donor/recipient CMV serostatus also differed between cohorts (p = 0.003): in the desensitization group, none were donor–recipient seronegative (−/−), ten (76.9%) were donor-negative/recipient-positive (−/+), none were donor-positive/recipient-negative (+/−), and three (23.1%) were donor–recipient seropositive (+/+), whereas in the non-desensitization group five (34.0%) were −/−, fourteen (29.8%) were −/+, eight (17.0%) were +/−, and nine (19.1%) were +/+. This difference is illustrated in Figure 1B, where CMV infection-free survival was significantly worse in the desensitization group (p < 0.0001). The desensitization group experienced a higher and earlier incidence of CMV infections following transplantation. Positive aspergillus galactomannan antigen tests tended to be less frequent in the desensitization group, though the difference did not reach statistical significance (8.3% vs. 21.9%, p = 0.055). Additionally, blood culture positivity rates for bacterial infections (2.8% vs. 6.9%, p = 0.49) and fungal infections (5.6% vs. 3.3%, p = 0.37) were comparable between the two groups.

**TABLE 3:** Infection and rejection outcomes.

Variable	Desensitization protocol (n = 36)	No Desensitization protocol (n = 363)	p value
Any infection	25 (69.4%)	223 (61.9%)	0.47
Respiratory infection	18 (50.0%)	188 (52.2%)	0.86
Recurrence respiratory infection	7 (19.4%)	59 (16.4%)	0.64
CMV infection	13 (36.1%)	47 (13.1%)	0.0009
Donor/Recipient CMV status			0.003
−/−	-	16 (34.0%)	
−/+	10 (76.9%)	14 (29.8%)	
+/−	-	8 (17.0%)	
+/+	3 (23.1%)	9 (19.1%)	
Positive aspergillus galactomannan antigen	3 (8.3%)	79 (21.9%)	0.055
Blood culture positive			
bacterial	1 (2.8%)	25 (6.9%)	0.49
fungal	2 (5.6%)	12 (3.3%)	0.37
ACR	6 (16.7%)	95 (26.4%)	0.23
number of ACR episodes	1.0 (1.0–2.0)	1.0 (1.0–2.0)	0.74
AMR	8 (22.2%)	12 (3.3%)	0.0001
number of AMR episodes	1.0 (1.0–2.5)	1.0 (1.0–1.0)	0.51
CLAD	5 (13.9%)	50 (13.8%)	1.00
BOS	2 (5.6%)	39 (10.7%)	0.56
RAS/mixed	3 (8.3%)	11 (3.0%)	0.12
Endpoint	Group	Median Event-Free Days (95% CI)	Log-rank p
ACR-free survival	Desensitized	NA (NA–NA)	
	No Desensitization	NA (NA–NA)	0.20
AMR-free survival	Desensitized	NA (NA–NA)	
	No Desensitization	NA (NA–NA)	<0.0001
Respiratory infection-free survival	Desensitized	184 (91–NA)	
	No Desensitization	274 (189–468)	0.93
CMV-free survival	Desensitized	NA (417–NA)	
	No Desensitization	NA (NA–NA)	<0.0001

Data are shown as number (%). CMV, Cytomegalovirus. *over 3 infections per year lasting over 4 weeks. ACR, acute cellular rejection; AMR, Antibody-mediated rejection; CLAD, chronic lung allograft dysfunction;BOS, bronchiolitis obliterans syndrome; RAS, restrictive allograft syndrome. Unknown date were excluded. NA, indicates that fewer than 50% of patients in that group experienced the event during follow-up, so the median event-free time is not reached.

### CMV Infection Incidence and Risk Within 1 Year

Supplementary Table S2 shows that, over a uniform one-year follow-up, CMV infection occurred in 9 of 36 patients (25.0%) who received perioperative desensitization versus 33 of 363 (9.1%) who did not (p = 0.007). Within the desensitized cohort, none of the nine CMV-infected patients were donor-negative/recipient-negative or donor-negative/recipient-positive; three (33.3%) were donor-positive/recipient-negative and six (66.7%) were donor-positive/recipient-positive. By contrast, among the 33 infected patients in the non-desensitized cohort, five (15.2%) were −/−, ten (30.3%) were −/+, seven (21.2%) were +/−, and 11 (33.3%) were +/+ (p = 0.003). In logistic regression—including mismatch status, perioperative desensitization protocol, and their interaction—the mismatch effect was not significant (OR 0.69, 95% CI 0.29–1.47; p = 0.36), whereas desensitization independently increased CMV risk more than fourfold (OR 4.23, 95% CI 1.90–9.20; p < 0.001). The interaction term yielded an OR effectively 0.00 (95% CI not estimable; p = 0.98), indicating no synergistic effect between mismatch and desensitization on CMV incidence (Supplementary Table S3).

### Rejection Outcomes

**FIGURE 2:**
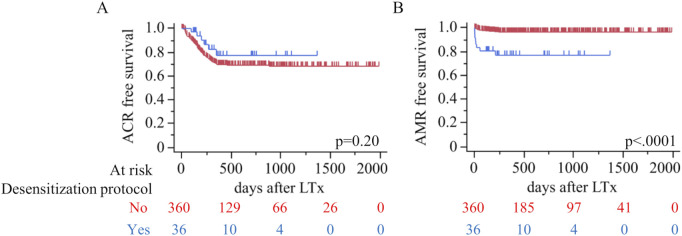
Rejection outcomes by desensitization status. Kaplan–Meier estimates of **(A)** ACR-free survival and **(B)** AMR-free survival after LTx (red, No; blue, Yes). Log-rank p-values are shown on each panel; tick marks indicate censoring. Numbers at risk are provided below the x-axis.

Rejection outcomes demonstrated no significant difference in the incidence of ACR between the desensitization and non-desensitization groups (16.7% vs. 26.4%, p = 0.23) (Table 3). The median number of ACR episodes was also similar between the groups [1.0 (1.0–2.0) vs. 1.0 (1.0–2.0), p = 0.74]. Figure 2A further illustrates ACR-free survival, showing no significant difference in survival rates between the two groups (p = 0.20). In contrast, AMR was significantly more frequent in the desensitization group compared to the non-desensitization group (22.2% vs. 3.3%, p = 0.0001). Although the median number of AMR episodes was comparable between the groups [1.0 (1.0–2.5) vs. 1.0 (1.0–1.0), p = 0.51], Figure 2B reveals a significantly worse AMR-free survival in the desensitization group (p < 0.0001). The desensitization group showed a higher and earlier incidence of AMR events following lung transplantation, no case of AMR persisted after corticosteroid pulse therapy and repeat TBLB confirmed histologic resolution. No difference was observed in CLAD between desensitization and non-desensitization groups.

### Predictors of PGD

Table 4 shows the risk factors associated with PGD of grade 2 or higher. Univariate logistic regression analysis identified several variables significantly associated with PGD. Among recipient factors, higher body mass index (BMI) was associated with an increased risk of PGD [odds ratio (OR) 1.05, 95% confidence interval (CI) 1.00–1.11, p = 0.037]. Bilateral lung transplantation (OR 1.67, 95% CI 1.05–2.70, p = 0.032) and higher PRA levels (OR 1.71, 95% CI 1.10–5.67, p = 0.017) were also significant predictors. Regarding etiology, pulmonary arterial hypertension (PAH) (OR 2.53, 95% CI 1.10–5.76, p = 0.029) and COVID-19-related indications (OR 2.32, 95% CI 1.23–4.31, p = 0.0094) were associated with increased PGD risk. Laboratory results showed that lower hemoglobin levels (OR 0.91, 95% CI 0.84–0.99, p = 0.037) and albumin levels (OR 0.60, 95% CI 0.40–0.91, p = 0.016) were significant predictors. Additionally, arterial oxygen pressure (PaO2) (OR 1.00, p = 0.028) and intraoperative factors, including operative time (OR 1.20, 95% CI 1.07–1.33, p = 0.0010), pRBC transfusion (OR 1.07, 95% CI 1.03–1.15, p = 0.0012),FFP transfusion (OR 1.11, 95% CI 1.02–1.21, p = 0.014), and VA-ECMO use (OR 1.85, 95% CI 1.16–3.01, p = 0.0096), were significant.

**TABLE 4:** Univariate and multivariate cox proportional hazard analysis as a predictor of PGD.

Variable	Univariate analysis			Multivariate analysis		
	Hazard Ratio	95% CI	p value	Hazard Ratio	95% CI	p value
Recipient factors						
Age, years	0.98	0.97–1.00	0.051			
Female	1.31	0.84–2.03	0.24			
BMI, kg/m2*	1.05	1.00–1.11	0.037	1.08	1.02–1.14	0.0072
BSA, m2*	1.89	0.78–4.63	0.16			
Smoking history	1.07	0.69–1.66	0.75			
Hypertension	1.15	0.74–1.79	0.55			
Diabetes	0.93	0.57–1.49	0.76			
CKD	1.13	0.50–2.39	0.77			
Bilateral	1.67	1.05–2.70	0.032	1.09	0.55–2.19	0.80
PRA	1.71	1.10–5.67	0.017	1.64	1.02–2.65	0.042
preformed DSA	1.86	0.99–3.41	0.053			
Desensitization protocol	1.72	0.83–3.46	0.14			
Etiology						
ILD	0.78	0.49–1.23	0.28			
COPD	0.53	0.27–0.97	0.038	0.82	0.41–1.64	0.57
PAH	2.53	1.10–5.76	0.029	2.39	0.98–5.84	0.056
COVID-19	2.32	1.23–4.31	0.0094	1.20	0.54–2.69	0.66
Laboratory						
Hemoglobin, g/dL*	0.91	0.84–0.99	0.037	1.03	0.92–1.17	0.60
WBC, 1,000/mm3*	1.03	0.97–1.09	0.34			
Platelets, 1,000/mm3*	1.00	1.00–1.00	0.86			
Sodium, mEq/L	1.03	0.97–1.10	0.32			
BUN, mg/dL	1.01	0.98–1.03	0.43			
Creatinine, mg/dL	1.39	0.59–3.24	0.45			
ALT, U/L*	1.00	0.99–1.01	0.93			
AST, U/L*	1.00	0.99–1.02	0.46			
Albumin, g/dL*	0.60	0.40–0.91	0.016	0.61	0.38–0.98	0.043
Total bilirubin, mg/dL	1.20	0.81–1.73	0.34			
INR	2.43	0.81–8.04	0.12			
Arterial blood gas						
pH	2.63	0.11–65.75	0.55			
PaCO2	1.01	0.99–1.02	0.48			
PaO2	1.00	1.00–1.00	0.028	1.00	1.00–1.00	0.74
Donor						
Age, years	1.02	1.00–1.04	0.052			
Female	1.21	0.76–1.91	0.43			
Intraoperative outcome						
Operative time (hours)	1.20	1.07–1.33	0.0010	1.03	0.86–1.23	0.75
Intra-op blood transfusion (unit)						
pRBC	1.07	1.03–1.15	0.0012	1.08	0.94–1.25	0.25
FFP	1.11	1.02–1.21	0.014	0.96	0.80–1.15	0.69
Plt	1.17	1.00–1.37	0.050			
Ischemic time (hours)	1.04	0.98–1.11	0.19			
VA-ECMO use	1.85	1.16–3.01	0.0096	1.27	0.68–2.37	0.46

BMI, body mass index; BSA, body surface area; CKD, chronic kidney disease; PRA, panel reactive antibody; DSA, donor specific antibody; ILD; interstitial lung disease; COPD, chronic obstructive pulmonary disease; PAH, pulmonary arterial hypertension; WBC, white blood cell; BUN, blood urea nitrogen; AST, aspartate aminotransferase; ALT, alanine aminotransferase; INR, international normalized ratio; pRBC, packed red blood cells; FFP, fresh frozen plasma; Plt, platelets; VA ECMO, veno-arterial extracorporeal membrane oxygenation. *Unknown cases were excluded.

In the multivariate analysis, higher BMI (OR 1.08, 95% CI 1.02–1.14, p = 0.0072) and PRA (OR 1.64, 95% CI 1.02–2.65, p = 0.042) remained significant independent predictors of PGD. Lower albumin levels were also independently associated with PGD (OR 0.61, 95% CI 0.38–0.98, p = 0.043).

### Predictors of CLAD

Table 5 presents both univariate and multivariate Cox proportional-hazards analyses for CLAD. In the univariate models, only two variables emerged as significant predictors: each 1 kg/m^2^ increase in BMI conferred an 8% higher hazard of CLAD (HR 1.08, 95% CI 1.02–1.15; p = 0.01), and each 1 g/dL rise in pre-transplant hemoglobin was associated with a 14% increase in risk (HR 1.14, 95% CI 1.02–1.27; p = 0.02). In contrast, variables such as PGD ≥2, CMV infection, and acute antibody-mediated rejection showed no significant univariate associations. In the fully adjusted multivariate model—including desensitization protocol, PGD ≥2, CMV, AMR, and recipient age—none of these factors remained independently significant. The desensitization protocol itself carried an adjusted hazard ratio of 1.75 (95% CI 0.67–4.57; p = 0.26), indicating that, after controlling for established risk factors, desensitization did not independently influence CLAD development.

**TABLE 5:** Univariate and multivariate cox proportional hazard analysis as a predictor of CLAD.

Variable	Univariate analysis			Multivariate analysis		
	Hazard Ratio	95% CI	p value	Hazard Ratio	95% CI	p value
Recipient factors						
Age, years	0.99	0.97–1.01	0.16	0.99	0.97–1.01	0.24
Female	0.84	0.49–1.46	0.55			
BMI, kg/m2*	1.08	1.02–1.15	0.01			
BSA, m2*	2.53	0.81–7.96	0.11			
Smoking history	1.16	0.68–1.98	0.58			
Hypertension	1.03	0.61–1.75	0.92			
Diabetes	1.38	0.80–2.39	0.25			
CKD	1.33	0.52–3.40	0.54			
Bilateral	0.84	0.49–1.46	0.54			
PRA	0.95	0.55–1.64	0.86			
preformed DSA	1.28	0.61–2.72	0.51			
Desensitization protocol	1.80	0.71–4.55	0.21	1.75	0.67–4.57	0.26
Etiology						
ILD	0.94	0.54–1.64	0.82			
COPD	1.07	0.76–1.48	0.71			
PAH	0.99	0.72–1.36	0.95			
COVID-19	0.97	0.80–1.17	0.74			
Laboratory						
Hemoglobin, g/dL*	1.14	1.02–1.27	0.02			
WBC, 1,000/mm3*	0.98	0.92–1.05	0.65			
Platelets, 1,000/mm3*	1.00	1.00–1.00	0.91			
Sodium, mEq/L	0.99	0.91–1.07	0.72			
BUN, mg/dL	1.01	0.98–1.04	0.36			
Creatinine, mg/dL	1.48	0.46–4.74	0.51			
ALT, U/L*	1.00	0.99–1.02	0.71			
AST, U/L*	1.00	0.98–1.01	0.73			
Albumin, g/dL*	1.45	0.93–2.27	0.10			
Total bilirubin, mg/dL	1.19	0.81–1.75	0.38			
INR	1.07	0.26–4.36	0.92			
Arterial blood gas						
pH	0.15	0.00–6.34	0.32			
PaCO2	0.98	0.96–1.01	0.14			
PaO2	1.00	1.00–1.00	0.67			
Donor						
Age, years	1.01	0.99–1.03	0.50			
Female	0.78	0.43–1.42	0.42			
Intraoperative outcome						
Operative time (hours)	0.95	0.83–1.09	0.46			
Intra-op blood transfusion (unit)						
pRBC	0.97	0.90–1.04	0.42			
FFP	1.00	0.91–1.11	0.97			
Plt	0.94	0.75–1.17	0.57			
Ischemic time (hours)	1.01	0.87–1.18	0.85			
VA-ECMO use	0.98	0.57–1.68	0.93			
Postoperative outcomes						
*de novo* DSA	1.19	0.61–2.30	0.61			
PGD						
any grade	0.72	0.41–1.26	0.25			
grade>=2	0.73	0.37–1.41	0.35	0.63	0.31–1.23	0.20
grade3	0.69	0.25–1.93	0.48			
AKI	0.61	0.34–1.10	0.10			
Dialysis	0.76	0.30–1.91	0.55			
Respiratory infection	1.59	0.90–2.82	0.11			
Positive aspergillus galactomannan antigen	1.35	0.74–2.46	0.33			
CMV infection	1.01	0.45–2.23	0.99	0.88	0.39–1.97	0.75
ACR	1.54	0.90–2.66	0.12			
AMR	2.40	0.95–6.05	0.06	2.21	0.87–5.62	0.097

CLAD, chronic lung allograft dysfunction; BMI, body mass index; BSA, body surface area; CKD, chronic kidney disease; PRA, panel reactive antibody; DSA, donor specific antibody; ILD; interstitial lung disease; COPD, chronic obstructive pulmonary disease; PAH, pulmonary arterial hypertension; WBC, white blood cell; BUN, blood urea nitrogen; AST, aspartate aminotransferase; ALT, alanine aminotransferase; INR, international normalized ratio; pRBC, packed red blood cells; FFP, fresh frozen plasma; Plt, platelets; VA ECMO, veno-arterial extracorporeal membrane oxygenation; DSA, donor specific antibody; PGD, primary graft dysfunction; AKI, acute kidney injury; CMV, cytomegalovirus; ACR, acute cellular rejection; AMR, Antibody-mediated rejection. *Unknown cases were excluded.

### Overall Survival

**FIGURE 3:**
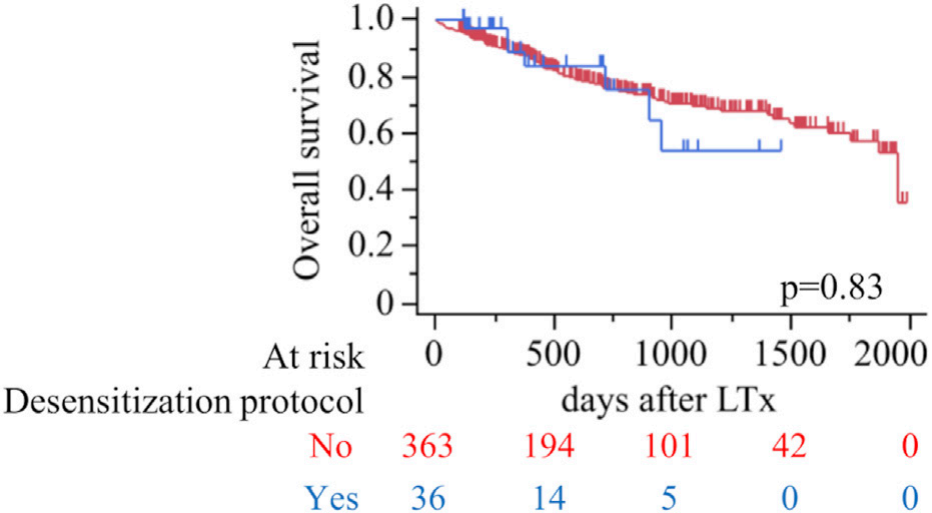
Overall survival after lung transplantation according to desensitization protocol (red, No; blue, Yes). Kaplan–Meier curves with numbers at risk shown below; tick marks indicate censoring. Log-rank p=0.83.

Figure 3 illustrates overall survival following lung transplantation for patients in the desensitization and non-desensitization groups. The Kaplan-Meier analysis revealed no significant difference in overall survival between the two groups (p = 0.83). The mean follow-up period for the cohort was 688.2 days (the sensitized group; 509.3 days, the desensitization group; 706.0 days). Additionally, Table 6 presents a univariate and multivariate cox proportional hazard analysis identifying predictors of overall survival. Significant findings from the multivariate analysis included bilateral lung transplantation (HR 0.44, 95% CI 0.28–0.69, p = 0.0004) and serum albumin levels (HR 0.14, 95% CI 0.030–0.64, p = 0.012), which were independently associated with improved survival. Postoperative outcomes such as PGD grade ≥2 (HR 1.76, 95% CI 1.11–2.80, p = 0.017), AKI (HR 1.82, 95% CI 1.15–2.88, p = 0.011), cerebrovascular accidents (CVA) (OR 3.48, 95% CI 1.33–9.09, p = 0.011), bowel ischemia (HR 3.04, 95% CI 1.06–8.71, p = 0.039), and digital ischemia (HR 6.48, 95% CI 2.45–17.11, p = 0.0002) were significant risk factors for reduced survival.

**TABLE 6:** Univariate and multivariate cox proportional hazard analysis as a predictor of overall survival.

Variable	Univariate analysis			Multivariate analysis		
	Hazard Ratio	95% CI	p value	Hazard Ratio	95% CI	p value
Recipient factors						
Age, years	1.01	0.99–1.03	0.26			
Female	1.07	0.71–1.61	0.74			
BMI, kg/m2*	1.04	0.99–1.08	0.12			
BSA, m2*	1.10	0.47–2.59	0.82			
Smoking history	1.05	0.70–1.57	0.81			
Hypertension	1.07	0.71–1.60	0.74			
Diabetes	1.40	0.93–2.12	0.11			
CKD	1.99	1.10–3.59	0.022	1.34	0.71–2.53	0.37
Bilateral	0.58	0.39–0.87	0.0078	0.44	0.28–0.69	0.0004
PRA	1.12	0.74–1.68	0.59			
preformed DSA	1.36	0.78–2.36	0.28			
Desensitization protocol	1.36	0.78–2.36	0.28			
Etiology						
ILD	1.09	0.72–1.65	0.69			
COPD	1.59	1.01–2.52	0.047	1.46	0.88–2.43	0.14
PAH	0.83	0.36–1.90	0.65			
COVID-19	0.77	0.41–1.45	0.41			
Laboratory						
Hemoglobin, g/dL*	1.00	0.92–1.08	0.94			
WBC, 1,000/mm3*	0.98	0.92–1.03	0.36			
Platelets, 1,000/mm3*	1.00	1.00–1.00	0.96			
Sodium, mEq/L	1.03	0.97–1.09	0.37			
BUN, mg/dL	1.55	0.14–12.06	0.70			
Creatinine, mg/dL	2.70	1.19–5.87	0.015			
ALT, U/L*	1.01	1.00–1.01	0.16			
AST, U/L*	1.01	1.00–1.01	0.073			
Albumin, g/dL*	0.18	0.035–0.95	0.043	0.14	0.030–0.64	0.012
Total bilirubin, mg/dL	1.17	0.85–1.50	0.26			
INR	1.25	0.41–3.14	0.67			
Arterial blood gas						
pH	0.14	0.0087–2.54	0.18			
PaCO2	1.00	0.98–1.01	0.70			
PaO2	1.00	1.00–1.00	0.65			
Donor						
Age, years	1.01	1.00–1.03	0.097			
Female	1.16	0.76–1.77	0.48			
Intraoperative outcome						
Operative time (hours)	0.97	0.87–1.07	0.53			
Intra-op blood transfusion (unit)						
pRBC	1.00	0.94–1.04	0.88			
FFP	1.01	0.92–1.08	0.89			
Plt	1.03	0.88–1.17	0.65			
Ischemic time (hours)	0.93	0.81–1.04	0.24			
VA-ECMO use	1.10	0.73–1.67	0.65			
Postoperative outcomes						
*de novo* DSA	0.8	0.45–1.42	0.45			
PGD						
any grade	1.49	0.99–2.26	0.056			
grade>=2	1.98	1.31–2.98	0.0012	1.76	1.11–2.80	0.017
grade3	3.33	2.12–5.25	<0.0001			
AKI	2.24	1.48–3.37	0.0001	1.82	1.15–2.88	0.011
Dialysis	3.25	2.10–5.05	<0.0001			
CVA	3.19	1.29–7.89	0.012	3.48	1.33–9.09	0.011
Bowel ischemia	11.31	4.51–28.36	<0.0001	3.04	1.06–8.71	0.039
Digital ischemia	5.74	2.32–14.18	0.0002	6.48	2.45–17.11	0.0002

BMI, body mass index; BSA, body surface area; CKD, chronic kidney disease; PRA, panel reactive antibody; DSA, donor specific antibody; ILD; interstitial lung disease; COPD, chronic obstructive pulmonary disease; PAH, pulmonary arterial hypertension; WBC, white blood cell; BUN, blood urea nitrogen; AST, aspartate aminotransferase; ALT, alanine aminotransferase; INR, international normalized ratio; pRBC, packed red blood cells; FFP, fresh frozen plasma; Plt, platelets; VA ECMO, veno-arterial extracorporeal membrane oxygenation; DSA, donor specific antibody; PGD, primary graft dysfunction; AKI, acute kidney injury; CVA, cerebrovascular attack. *Unknown cases were excluded.

## Discussion

In this study, we examined the outcomes of sensitized patients who underwent lung transplantation following the desensitization protocol with eculizumab, comparing their perioperative and postoperative courses to those of non-sensitized patients. Several key findings emerged [1]: despite receiving intensified immunosuppressive therapy, the desensitization group had comparable one-year and overall survival rates [2]; the incidence of infections was broadly similar between the two groups, except a significantly higher rate of CMV infection in the desensitization group [3], ACR and CLAD rates did not differ significantly, yet AMR was more frequent in the desensitization group, and [4] although the desensitization group experienced longer ICU stays and required more intraoperative transfusions, their one-year graft and patient survival remained comparable to non-sensitized controls. These findings suggest that lung transplantation can be performed safely in sensitized recipients when the desensitization protocol with eculizumab is implemented alongside meticulous postoperative monitoring. One of the critical observations of this study is the increased incidence of AMR in the desensitization group. The presence of pre-formed DSAs and the resultant immunological milieu likely account for this higher incidence. Despite the heightened risk of AMR, rigorous triple immunosuppressive management (Tacrolimus, prednisone, and mycophenolate) and close clinical monitoring contributed to controlling these episodes and preventing detrimental effects on graft function and patient survival. This underscores that while desensitization can enable transplantation in sensitized patients, it necessitates vigilant post-transplant follow-up to detect and treat rejection promptly.

Our results demonstrate that lung transplantation in sensitized patients with desensitized protocol including eculizumab is feasible and can yield survival rates comparable to those of non-sensitized patients. Historically, the presence of pre-formed DSAs has been a major concern, as it predisposes recipients to early graft failure or hyperacute rejection [17, 18]. However, advances in immunosuppression, plasmapheresis, and targeted biological therapies have paved the way for more aggressive desensitization protocols [19]. Our data align with emerging evidence from other centers, which likewise suggests that, while sensitized patients carry an elevated risk profile, this risk does not necessarily translate into inferior overall survival if managed appropriately [20–22]. The finding that one-year and overall survival did not differ significantly between the two groups is particularly noteworthy. This indicates that the immunologic risks that are traditionally associated with high sensitization status may be mitigated through our specialized perioperative and postoperative regimens including eculizumab. Such findings are significant for transplant centers that may otherwise exclude sensitized patients from lung transplant candidacy, offering a viable approach to expand access to transplantation for this challenging population. Importantly, our desensitization regimen is unique in two respects. First, we employ perioperative complement inhibition with eculizumab (C5 blockade), a strategy pioneered in kidney transplantation to prevent antibody-mediated injury but not previously reported in large lung transplant cohorts. Second, we combine interleukin-2 receptor blockade (basiliximab) and polyclonal T-cell depletion (rabbit ATG) during induction—agents that are normally used as alternatives but here are used synergistically to blunt both cellular and humoral alloimmunity. While these intensifications carry a theoretical increased risk of opportunistic infections and cytopenias, our data show that CMV and other infection rates remain manageable (see Table 3; Supplementary Table S3), and no cases of refractory AMR were observed. Taken together, the marked reduction in early AMR and the preservation of one-year and overall survival suggest that the benefits of this two-pronged, complement-targeted approach outweigh the risks in this high-risk, sensitized population. One caveat of our approach is that therapeutic plasma exchange (PLEX) can remove circulating eculizumab, since the monoclonal antibody is itself an IgG. In our protocol we therefore administer eculizumab immediately after each PLEX session to partially offset this loss, but studies in other fields estimate that a single PLEX can clear 40%–60% of infused antibody. As a result, trough complement activity may transiently rebound between exchange and dosing. Although we did not measure CH_50_ or free eculizumab levels in this series, future work should incorporate pharmacodynamic monitoring to optimize the timing and dosing of eculizumab around PLEX and ensure continuous complement blockade.

One of the more concerning complications in sensitized patients is the potential for AMR, which could lead to CLAD development. Our study revealed that AMR occurred significantly more frequently in the desensitization group (22.2% vs. 3.3%, p = 0.0001), consistent with pre-existing DSAs that can drive humoral immune responses against graft. Given these patients’ substantial immunologic burden, it is not entirely surprising that AMR rates were elevated even though perioperative desensitized protocol. However, despite the higher frequency of AMR events, these episodes were manageable with augmented immunosuppression and close clinical follow-up, preventing a negative impact on CLAD rate and overall survival. In contrast, ACR rates did not differ significantly between the two groups. This implies that the cellular immunologic pathways underlying ACR may be effectively controlled by standard immunosuppressive regimens, which typically include calcineurin inhibitors and anti-proliferative agents alongside steroids. The heightened concern for AMR in this subset reinforces the need for close surveillance of DSA titers and incorporating protocolized biopsies to ensure timely detection and intervention.

Given the potent immunosuppressive therapies employed, an essential aspect of managing sensitized patients is balancing the risk of rejection against the risk of infection [5]. In our cohort, the overall incidence of infections, excluding cytomegalovirus, was not significantly different between the desensitization and non-desensitization groups. Respiratory infections, including bacterial pneumonia and recurrent infections, were similarly frequent, suggesting that the standard infection prophylaxis regimens are effective in both populations. However, CMV infections were notably more common in the desensitization group. As shown in Supplementary Table S3, multivariable logistic regression demonstrated that the desensitization protocol independently increased CMV infection risk more than fourfold (OR 4.23, 95% CI 1.90–9.20; p < 0.001), whereas serologic mismatch itself was not a significant predictor (OR 0.69, 95% CI 0.29–1.47; p = 0.36). The interaction term between mismatch and desensitization was also non-significant (OR effectively 0.00; p = 0.98), indicating that intensified immunosuppression, rather than mismatch status, drives the elevated CMV risk across all desensitized patients. This higher incidence reflects the intensified immunosuppressive approach and the frequent use of additional agents, such as eculizumab and anti-thymocyte globulin, which further compromise antiviral immunity. Early onset of CMV infection in these patients (median onset at 262.0 days post-transplantation, IQR: 97.8–401 days) underscores the importance of robust CMV surveillance strategies, which may include routine viral load monitoring, prophylactic or preemptive antiviral therapy, and meticulous follow-up. Previously, we reported that CMV infection remains a critical complication in lung transplant recipients, particularly those with serological mismatch [23]. At our center, CMV prophylaxis is routinely administered for up to 1 year post-transplantation, utilizing valganciclovir as the primary agent. This protocol has significantly reduced CMV-related morbidity; however, the risk of late-onset CMV infection following the cessation of prophylaxis persists. Based on our previous study, the median onset of CMV infection after lung transplantation was reported to occur at approximately 395 days (IQR: 264–453) for all patients and at 425 days (IQR: 405–456) after completing prophylaxis in serological mismatch cases. The recurrence rate highlights the importance of tailoring CMV management strategies, particularly in high-risk cohorts. Encouragingly, while CMV infections were more frequent, this did not compromise overall survival, suggesting that aggressive diagnosis and treatment protocols can mitigate most adverse outcomes. Moreover, the fact that other infection rates—such as aspergillus galactomannan antigen positivity, bacteremia, and fungal bloodstream infections—remained similar between groups indicates that the increased susceptibility is largely CMV-specific, supporting targeted adjustments to CMV prevention rather than broad-spectrum antimicrobial changes. It is also worth noting that other infection rates—such as aspergillus galactomannan antigen positivity, bacteremia, or fungal bloodstream infections—did not differ significantly. This finding reassures that enhanced immunosuppression in desensitized patients may not universally increase susceptibility to all pathogens but rather select agents like CMV.

A noteworthy point in our analysis is the higher use of VA-ECMO intraoperatively in the desensitization group compared to the non-desensitization group. This could reflect either a preference for more aggressive intraoperative support in patients perceived to be at higher risk or an actual clinical necessity due to their heightened perioperative instability. VA-ECMO use could introduce risks such as bleeding, thrombotic events, and inflammatory cascade activation that might contribute to PGD [24, 25]. Interestingly, PGD severity at 72 h did not differ significantly between the groups, though there was a trend toward higher PGD grades in the desensitization group. Prior literature has consistently identified both donor- and recipient-related factors contributing to PGD, including high BMI, pulmonary hypertension, and the presence of DSAs [26, 27]. The association of bilateral transplantation with increased PGD likely reflects the greater surgical insult, longer ischemic times, and higher transfusion requirements inherent to bilateral procedures. Conversely, bilateral grafts confer superior long-term pulmonary mechanics, ventilation–perfusion matching, and reserve—factors that ultimately translate into a survival advantage despite a higher early PGD risk. Thus, the short-term vulnerability to reperfusion injury does not negate the medium- and long-term benefit of bilateral allografts. Multivariate analysis in our study confirmed that higher BMI, elevated PRA, and lower albumin levels were independent predictors of PGD. While our data do not definitively implicate the desensitization protocol with eculizumab alone as a driver of PGD, sensitized patients may come to transplants with more challenging clinical profiles overall.

The findings of this study reinforce the notion that sensitized patients can undergo successful lung transplantation if adequately managed. The elevated risk of AMR, CMV infection, and additional resource utilization does not appear to compromise long-term survival. Thus, the standard of care may evolve to include routine evaluation of patients previously excluded solely based on high sensitization statuses. In comparison to studies using alternative desensitization strategies, such as the protocol described by Aversa et al [8], our protocol—with the addition of eculizumab—represents a more aggressive immunosuppressive approach. Importantly, the critical role of complement activation in graft injury strongly supports the use of eculizumab. Evidence from our prior study, demonstrated a clear temporal correlation between post-reperfusion complement deposition and severe primary graft dysfunction in lung allografts [28]. This finding underscores that complement-mediated injury is a key driver of graft dysfunction in this setting, making complement inhibition not merely an adjunct but an essential therapeutic component.

Several limitations should be acknowledged. First, this was a single-center cohort study, and the desensitization protocol with eculizumab may not be universally implemented or standardized. Protocol variations across centers could result in different outcomes, thus limiting the generalizability of our findings. Second, although our analysis included a substantial number of lung transplants, the proportion of desensitized patients was relatively small, reflecting the lower prevalence of sensitized candidates. This disparity may introduce some statistical limitations in detecting small but meaningful differences. Third, we did not collect systematic post-transplant DSA clearance data beyond routine monthly surveillance, so we cannot directly correlate pfDSA kinetics with clinical outcomes. Fourth, we did not perform a comprehensive cost-effectiveness evaluation. However, it is clear from our results that the desensitization group incurred higher resource utilization, at least in terms of transfusions and possibly extended ICU stays. Finally, our median follow-up was shorter in the desensitized cohort (367 vs. 567 days; p = 0.06), which may limit the detection of CLAD—an outcome that typically accumulates over several years. Longer follow-up will therefore be required to fully assess the impact of desensitization on long-term CLAD risk. In addition, our institutional protocol used PRA >40% as the threshold for initiating desensitization, regardless of the presence of preformed DSA. While this approach maximized safety, it may have resulted in overtreatment of patients without pfDSA. Future protocols may refine these criteria to target desensitization more precisely. Another important limitation is the absence of repeated IVIG maintenance infusions in our protocol, which may have contributed to the higher incidence of AMR observed in the desensitized group.

In conclusion, our study supports that lung transplantation in sensitized patients is feasible and safe with appropriate desensitization protocols and vigilant postoperative care. However, these patients are at higher risk for certain complications—most notably AMR and CMV infections—and their overall survival rates are comparable to non-sensitized recipients. Future research directions include multi-institutional trials to validate our findings and further refine desensitization protocol, investigate long-term graft function beyond the first year, and develop biomarkers or diagnostic tools to detect impending AMR earlier. Ultimately, our results underscore the importance of expanding lung transplant eligibility to include sensitized patients who can benefit substantially from transplantation when managed with an optimized, individualized immunosuppressive approach.

## Data Availability

The data presented in this study are available on reasonable request by a qualified investigator for three years after the date of publication from the corresponding author.

## References

[1] Meyer KC. Recent Advances in Lung Transplantation. London, United Kingdom: F1000 Research Ltd. (2004) 7. 10.12688/f1000research.15393.1 PMC6206601 30416706

[2] Davidson BT Donaldson TA. Immune System Modulation in the Highly Sensitized Transplant Candidate. Crit Care Nurs Q (2004) 27:1–9. 10.1097/00002727-200401000-00001 14974520

[3] Iyer HS Jackson AM Montgomery RA. Sensitized Patients, Transplant, and Management. Curr Transpl Rep (2014) 1(2):69–77. 10.1007/s40472-014-0010-0

[4] Kahwaji J Choi J Vo A Jordan SC. Immunologic and Infectious Complications in Highly Sensitized Patients Post-kidney Transplantation. Clin Transpl (2015) 31:265–73. 28514588

[5] Habli M Belal D Sharma A Halawa A. Transplanting Highly Sensitized Patients. J The Egypt Soc Nephrol Transplant (2023) 23(2):45–52. 10.4103/jesnt.jesnt_34_22

[6] Sharma A King A Kumar D Behnke M McDougan F Kimball PM. Perioperative Desensitization Improves Outcomes Among Crossmatch Positive Recipients of Deceased Donor Renal Transplants. Prog Transplant (2016) 26(2):157–61. 10.1177/1526924816640678 27207404

[7] Kuppachi S Axelrod DA. Desensitization Strategies: Is It Worth It?. Transpl Int (2020) 33. 251–9. 10.1111/tri.13532 31553805

[8] Aversa M Martinu T Patriquin C Cypel M Barth D Ghany R Long-Term Outcomes of Sensitized Lung Transplant Recipients After peri-operative Desensitization. Am J Transplant (2021) 21(10):3444–8. 10.1111/ajt.16707 34058795

[9] Tinckam KJ Keshavjee S Chaparro C Barth D Azad S Binnie M Survival in Sensitized Lung Transplant Recipients with Perioperative Desensitization. Am J Transplant (2015) 15(2):417–26. 10.1111/ajt.13076 25612494

[10] Aversa M Kiernan J Martinu T Patriquin C Barth D Li Q Outcomes After Flow Cytometry crossmatch-positive Lung Transplants Managed with Perioperative Desensitization. Am J Transplant (2023) 23(11):1733–9. 10.1016/j.ajt.2023.04.033 37172694

[11] Parquin F Zuber B Vallée A Taupin JL Cuquemelle E Malard S A Virtual Crossmatch-based Strategy for Perioperative Desensitisation in Lung Transplant Recipients with Pre-formed Donor-specific Antibodies: 3-Year Outcome. Eur Respir J (2021) 58(5):2004090. 10.1183/13993003.04090-2020 34016620

[12] Heise EL Chichelnitskiy E Greer M Franz M Aburahma K Iablonskii P Lung Transplantation Despite Preformed Donor-Specific Antihuman Leukocyte Antigen Antibodies: A 9-Year Single-Center Experience. Am J Transplant (2023) 23(11):1740–56. 10.1016/j.ajt.2023.04.034 37225088

[13] Marfo K Lu A Ling M Akalin E. Desensitization Protocols and Their Outcome. Clin J Am Soc Nephrol (2011) 6(4):922–36. 10.2215/CJN.08140910 21441131

[14] Stegall MD Diwan T Raghavaiah S Cornell LD Burns J Dean PG Terminal Complement Inhibition Decreases Antibody-Mediated Rejection in Sensitized Renal Transplant Recipients. Am J Transplant (2011) 11(11):2405–13. 10.1111/j.1600-6143.2011.03757.x 21942930

[15] Coutance G Kobashigawa JA Kransdorf E Loupy A Desiré E Kittleson M Intermediate-Term Outcomes of Complement Inhibition for Prevention of Antibody-Mediated Rejection in Immunologically High-Risk Heart Allograft Recipients. J Heart Lung Transplant (2023) 42(10):1464–8. 10.1016/j.healun.2023.05.005 37182818

[16] Thomae BL Kaihou T Pinelli DF Friedewald JJ Caicedo-Ramirez JC Bharat A Successful Multiorgan Transplantation in Highly Sensitized Patients with Positive Crossmatch Donor. Clin Transpl (2024) 38(12):e70040. 10.1111/ctr.70040 39620859

[17] Redondo-Pachón D Pérez-Sáez MJ Mir M Gimeno J Llinás L García C Impact of Persistent and Cleared Preformed HLA DSA on Kidney Transplant Outcomes. Hum Immunol (2018) 79(6):424–31. 10.1016/j.humimm.2018.02.014 29524568

[18] O’Leary JG Kaneku H Jennings LW Bañuelos N Susskind BM Terasaki PI Preformed Class II Donor-specific Antibodies Are Associated With An Increased Risk of Early Rejection After Liver Transplantation. Liver Transplant (2013) 19(9):973–80. 10.1002/lt.23687 23780820

[19] Snyder LD Gray AL Reynolds JM Arepally GM Bedoya A Hartwig MG Antibody Desensitization Therapy in Highly Sensitized Lung Transplant Candidates. Am J Transplant (2014) 14(4):849–56. 10.1111/ajt.12636 24666831 PMC4336170

[20] Montgomery RA Lonze BE King KE Kraus ES Kucirka LM Locke JE Desensitization in HLA-Incompatible Kidney Recipients and Survival. New Engl J Med (2011) 365(4):318–26. 10.1056/NEJMoa1012376 21793744

[21] Reinsmoen NL Lai CH Vo A Jordan SC. Evolving Paradigms for Desensitization in Managing Broadly HLA Sensitized Transplant Candidates. Discov Med (2012) 13:267–73. 22541614

[22] Noble J Metzger A Daligault M Chevallier E Bugnazet M Bardy B Immortal time-Bias–Corrected Survival of Highly Sensitized Patients and HLA-Desensitized Kidney Transplant Recipients. Kidney Int Rep (2021) 6(10):2629–38. 10.1016/j.ekir.2021.07.024 34622102 PMC8484495

[23] Toyoda T Kurihara C Kaiho T Arunachalam A Lysne J Thomae BL Predictors of Cytomegalovirus Recurrence Following Cessation of Posttransplant Prophylaxis. J Surg Res (2024) 299:129–36. 10.1016/j.jss.2024.04.012 38754251

[24] Kurihara C Manerikar A Querrey M Felicelli C Yeldandi A Garza-Castillon R Clinical Characteristics and Outcomes of Patients with COVID-19-Associated Acute Respiratory Distress Syndrome who Underwent Lung Transplant. JAMA (2022) 327(7):652–61. 10.1001/jama.2022.0204 35085383 PMC8796055

[25] Takahashi T Terada Y Pasque MK Nava RG Kozower BD Meyers BF Outcomes of Extracorporeal Membrane Oxygenation for Primary Graft Dysfunction After Lung Transplantation. Ann Thorac Surg (2023) 115(5):1273–80. 10.1016/j.athoracsur.2022.12.038 36634836

[26] Liu Y Liu Y Su L Jiang SJ. Recipient-Related Clinical Risk Factors for Primary Graft Dysfunction After Lung Transplantation: A Systematic Review and Meta-Analysis. PLoS One (2014) 9(3):e92773. 10.1371/journal.pone.0092773 24658073 PMC3962459

[27] Barr ML Kawut SM Whelan TP Girgis R Böttcher H Sonett J Report of the ISHLT Working Group on Primary Lung Graft Dysfunction Part IV: Recipient-Related Risk Factors and Markers. J Heart Lung Transplant (2005) 24(10):1468–82. 10.1016/j.healun.2005.02.019 16210118

[28] Cerier E Kurihara C Kaiho T Toyoda T Manerikar A Kandula V Temporal Correlation Between Postreperfusion Complement Deposition and Severe Primary Graft Dysfunction in Lung Allografts. Am J Transplant (2024) 24(4):577–90. 10.1016/j.ajt.2023.11.006 37977230 PMC10982049

