# Supplementary material for: Long Term Outcomes of Lung Transplantation in Sensitized Patients Following Eculizumab Use With the Desensitization Protocol
Source: Transpl Int. 2025 Sep 22;38:15040. doi: 10.3389/ti.2025.15040 (PMC12497660; doi:10.3389/ti.2025.15040)
Supplement: Supplementary file 1 [file DataSheet1.pdf]

Supplementary information for

Long term Outcomes of Lung Transplantation in Sensitized Patients Following Eculizmab Use with the Desensitization Protocol

Yudai Miyashita, Taisuke Kaiho, David F. Pinelli, Anthony Joudi, Mihir John, Austin Chang,

Benjamin Louis Thomae, Amanda Kamar, Carl Atkinson,

Ankit Bharat, GR Scott Budinger, Ambalavanan Arunachalam, Chitaru Kurihara

Corresponding Author:

Chitaru Kurihara, MD

E-mail; chitaru.kurihara@northwestern.edu

This PDF file includes:

Tables S1, S2 and S3

Figure S1

Supplemental Methods

Supplemental Table S1. Summary of positive crossmatch perioperative desensitization & induction

Day	Plasma Exchange	Eculizumab	Basiliximab	Methylprednisolone	rATG	IVIG
**Pre-operative**	Full FFP	1200 mg				300 mg/kg
**Intra-operative**			20 mg	500 mg		
**POD 0**	Full FFP	900 mg		0.4 mg/kg		
**POD 1**	Full FFP	600 mg		0.4 mg/kg		*300 mg/kg(If IgG < 500)
**POD 2**	Full FFP	600 mg		0.4 mg/kg		*300 mg/kg(If IgG < 500)
**POD 3**	50/50	1200 mg		0.4 mg/kg		*300 mg/kg(If IgG < 500)
**POD 4**			20 mg	0.4 mg/kg		1 g/kg
**POD 5**				125 mg	1 mg/kg	
**POD 6**				0.4 mg/kg	1 mg/kg	
**POD 7**				0.4 mg/kg	1 mg/kg	
**POD 8**				0.4 mg/kg	1 mg/kg	
**POD 9**				0.4 mg/kg	1 mg/kg	
**POD 10**				0.4 mg/kg	1 mg/kg	

Weekly STAT DSA checks are performed and prior to eculizumab infusions.

*Obtain donor-specific antibodies after plasma exchange and before IVIG administration

rATG, Rabbit Anti-Thymocyte Globulin; IVIG, Intravenous Immunoglobulin; POD,Postoperative Day; FFP, Fresh Frozen Plasma.

Supplemental Table S2. immunologic characteristics of the preformed DSA

anti-	number (ratio)
A	9 (25.0%)
B	18(50.0%)
C	8 (22.2%)
DR	8 (22.2%)
DQ	5 (13.9%)
DP	5 (13.9%)

Some patients had DSAs against multiple HLA loci; categories are not mutually exclusive.

Supplemental Table 3. One-Year CMV Infection and Antibody-Mediated Rejection Outcomes by Desensitization Protocol and Logistic Regression Analysis of CMV Infection (≤ 1 years)

Variable	Desensitization protocol (n=36)	No Desensitization protocol (n=363)	p value
CMV infection	9 (25.0%)	33 (9.1%)	0.007
Donor/Recipient CMV status			
-/-		5 (15.2%)	
-/+		10 (30.3%)	
+/-	3 (33.3%)	7 (21.2%)	
+/+	6 (66.7%)	11 (33.3%)	
AMR	8 (22.2%)	12 (3.3%)	0.0001
**Logistic regression (≤360 days)**	**OR**	**95% CI**	**p-value**
Mismatch (Yes vs No)	0.69	0.29-1.47	0.36
Desensitization (Yes vs No)	4.23	1.90-9.20	<0.001
Interaction (Mismatch × Desensit.)	0.00	not estimable	0.98

Data are shown as number (%). CMV, Cytomegalovirus; AMR, Antibody-mediated rejection. Unknown date were excluded.

Supplemental Figure S1

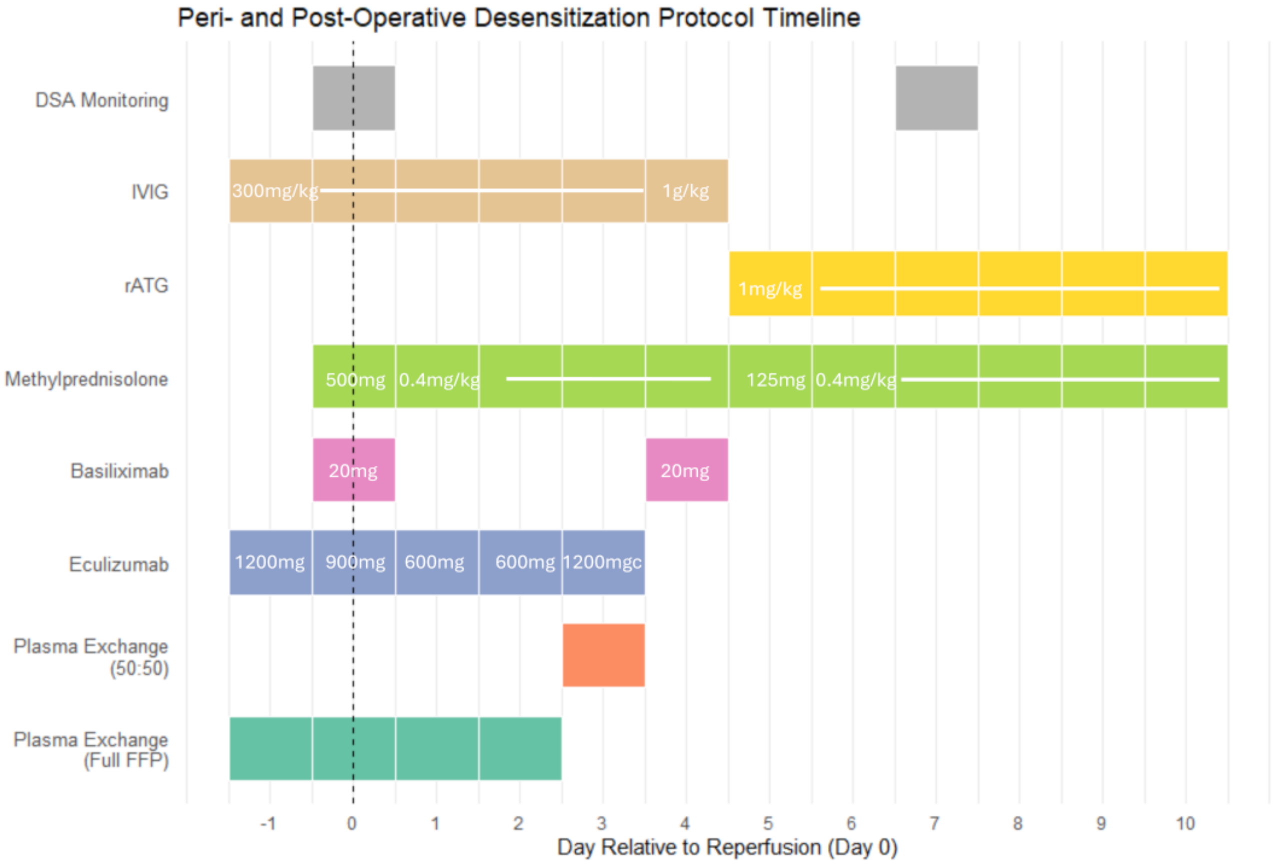

Supplemental Methods

Peri- and Post-Operative Desensitization Protocol for Sensitized Lung Transplant Recipients

Sensitized candidates—defined as those with a panel-reactive antibody (PRA) level ≥40% by Luminex single‑antigen bead assay—underwent an intensive peri-operative desensitization regimen. Detailed HLA testing (virtual crossmatch followed by prospective flow crossmatch when indicated) is outlined below. Key elements of the protocol are summarized in Supplemental Table S1, Figure S1 and included the following:

Plasma Exchange (PLEX)

One session 4–6 hours prior to graft reperfusion (full‑volume exchange with fresh frozen plasma)

Four additional sessions on postoperative days (POD) 0, 1, 2, and 3 (POD3 exchange volume split 50:50 between plasma and albumin)

Eculizumab

1,200 mg immediately pre-operatively

900 mg on POD 0

600 mg on POD 1 and 2

1,200 mg on POD 3

Induction Immunosuppression

Methylprednisolone: 1000 mg intra-operatively, then 20 mg IV once daily on POD 4

Basiliximab: 20 mg IV intra-operatively and on POD 4

Rabbit Anti-Thymocyte Globulin (rATG): 1 mg/kg/day IV on POD 5–10 (cumulative dose 4–8 mg/kg)

Intravenous Immunoglobulin (IVIG): 300 mg/kg IV on POD 0–3 if post-exchange serum IgG < 500 mg/dL

1 g/kg IV on POD 4. IVIG was administered only during the perioperative window (300 mg/kg on POD 0–3 if IgG < 500 mg/dL, and 1 g/kg on POD 4); no routine IVIG maintenance infusions were scheduled post-

discharge, and further IVIG was reserved for specific clinical indications.

Monitoring

Weekly DSA measurement by Luminex and complement - binding (C1q) assay

DSA sampled immediately before each eculizumab dose to assess clearance.

HLA Antibody Testing

All antibody testing was performed and reported at the time of treatment as part of routine clinical care. Serum was tested by FlowPRA Class I and II Screening test (One Lambda/ThermoFisher Scientific, Los Angeles, CA) on a Cytoflex flow cytometer (Beckman Coulter, Brea, CA). Antibody specificities were determined using LABScreen Single Antigen Class I and Class II Beads (One Lambda/ThermoFisher Scientific, Los Angeles, CA) on a Luminex 3D platform (Luminex Corporation, Austin, TX). Samples were tested at the time of initial evaluation by FlowPRA and Single Antigen Beads. Serum was collected immediately prior to transplant to use in the retrospective Flow Cytometry crossmatch. Post-transplant samples were tested by Single at various intervals depending on the patient, but protocol monitoring was performed monthly for the first year post-transplant, with additional samples at the time of bronchoscopy or signs of clinical dysfunction.

Immunosuppression management

Induction

- Methylprednisolone 1,000 mg via intravenous at intraoperatively.
- Basiliximab 20 mg at intraoperatively and POD 4.

Maintenance immunosuppression

- Prednisone 0.5 mg/kg p.o. daily from POD 1. Maximum dose = prednisone 40 mg daily. 0.5 mg/kg daily for 1 month, then taper by 5 mg every 2 weeks down to 5 mg/day as a maintenance dose.
- Mycophenolate mofetil 1,000 mg b.i.d. from POD 1.

- Tacrolimus start POD 1. Goal target levels 8–12 within first year post-transplant; then target 8–10 thereafter.

VV-ECMO indication criteria

Prior to lung transplantation, all intubated patients were treated by a multidisciplinary team in accordance with the guidelines of the National Heart, Lung, and Blood Institute's ARDS Network [1]. Indications for ECMO evaluation included refractory hypoxemia with PaO_2 less than 55 mmHg, pulse oximetry oxygen saturation less than 88%, and pH level less than 7.2. Patients were evaluated with lung-protective mechanical ventilation with a plateau pressure of less than 35 mmHg, neuromuscular blockade, and prone positioning, according to recommendations from the Extracorporeal Life Support Organization [2].

Anticoagulation during VV-ECMO support

Patients did not receive continuous anticoagulation unless there was a specific indication, such as deep venous thrombosis or pulmonary embolism, and there was no monitoring of bleeding parameters, such as activated clotting time or activated partial thromboplastin time. All patients not receiving continuous systemic anticoagulation received 5,000 U of subcutaneous unfractionated heparin every 8 hours as a prophylactic dose to prevent deep venous thrombosis. VV-ECMO flow was maintained at a minimum of 3.0-3.5 L/min, consistent with our recent reports, to reduce thrombotic complications in the ECMO circuit [3,4]

Intraoperative VA-ECMO Indications

We routinely employ VA-ECMO during lung transplantation to capitalize on several key advantages that enhance surgical and postoperative outcomes[5–10]. Specifically, VA-ECMO is indicated when:

- Hemodynamic Stability: It is required to maintain stability, especially when surgical access to the hilar structures is challenging.

- Lung-Protective Ventilation: It facilitates the use of lung-protective ventilation strategies by allowing for low inspired oxygen fractions and reduced driving pressures.
- Controlled Reperfusion: It ensures prolonged, controlled reperfusion of the newly implanted graft.
- Right Heart Support: It minimizes right ventricular strain, particularly during pulmonary artery clamping.

Surgical Technique

The patient was intubated using a double-lumen endotracheal tube. After standard line placement, immunosuppression and prophylactic antibiotics were administered. A standard clamshell incision for bilateral lung transplantation or a right or left anterolateral thoracotomy for single lung transplantation was performed, and the inferior pulmonary ligament was taken down. The upper and lower lobe pulmonary veins were carefully encircled and divided using a vascular stapler. The pulmonary artery (PA) was similarly encircled and divided using a vascular stapler. The bronchus was divided using a TA. Peripheral veno-arterial extracorporeal membrane oxygenation (VA ECMO) was initiated by cannulating the femoral artery and vein using an 18 French femoral cannula (Edwards Lifesciences, Irvine, CA, USA) and a 25-French Biomedicus cannula (Medtronic plc, Mounds View, MN, USA), respectively. The V-V ECMO circuit included Quadrox iD adult (7.0) oxygenator (MAQUET Holding B.V. & Co. KG, Germany) and Rotaflow pump (MAQUET Holding B.V. & Co. KG, Germany). Except for the cannulas the other components of the circuit had a heparin coating. The patient received 5,000 units of heparin, and the activated clotting time (ACT) was maintained between 200-250 seconds. The pericardium was opened, and a PA vent was placed using a 16-French femoral cannula, Y-connected into the drainage circuit. After initiating VA ECMO with the PA vent, a left pneumonectomy was performed in a similar manner to the right-side pneumonectomy. Following bilateral pneumonectomy, the double-lumen tube was exchanged for a single-lumen tube. Right lung implantation was performed using standard techniques, followed by removal of the PA vent. Left lung implantation was then completed using the same steps and suture materials. After both lung implants were successfully in place, the patient was weaned off VA ECMO, which was subsequently decannulated. The chest was closed in the standard fashion.

Infection Prophylaxis and CMV Monitoring Strategies

Pneumocystis jirovecii pneumonia prophylaxis was uniformly administered using Bactrim DS twice weekly (Mondays and Wednesdays).

CMV prophylaxis was tailored based on the serostatus risk stratification of donors and recipients. Specifically, patients with CMV R(+) D(+) and R(+) D(–) status received valganciclovir 900 mg orally once daily for 6 months, while those with CMV R(–) D(+) status were treated for 12 months[11]. In selected cases of CMV R(+) D(+) patients, a shorter regimen of valacyclovir (500 mg orally twice daily for 3 months) was employed depending on individual risk factors. Concurrent with these pharmacological interventions, our center implemented an intensive CMV surveillance protocol. Quantitative CMV PCR monitoring was performed every 1–2 weeks during the first three months post-transplant, then reduced to 2–4 weeks between months 3 and 12. More specifically, the schedule involved weekly measurements for the first 4 weeks, biweekly assessments during the second month, monthly monitoring over the following 9 months, and subsequent evaluations every three months. After discontinuation of valganciclovir at one-year, biweekly PCR testing was carried out for an additional month, with further monitoring reserved for symptomatic patients.

Fungal prophylaxis was ensured with posaconazole administered for 6 months, and in patients receiving eculizumab, meningococcal prophylaxis with Penicillin V for 3 months was instituted. Furthermore, all patients—regardless of sensitization status—received vaccination against meningococcal disease ideally at least 2 weeks prior to transplant listing, a measure particularly vital for sensitized patients on eculizumab to mitigate the risk of meningococcal infection.

Rejection Monitoring and Management

Cases fulfilling the diagnostic criteria for antibody-mediated rejection (AMR) as described below were treated with high-dose corticosteroid pulse therapy (methylprednisolone 500 mg IV daily for 3 days). One month after completion of pulse therapy, patients underwent repeat transbronchial lung biopsy (TBLB) to assess histologic response, and AMR was re-evaluated by C4d staining and DSA monitoring.

Definition of complication

Primary graft dysfunction (PGD)

PGD was defined based on the ISHLT guideline[12] , and graded by PaO2/FiO2 ratio as follows; Grade 1: PaO2/FiO2 ratio >300; Grade 2: PaO2/FiO2 ratio is 200-300; Grade 3: PaO2/FiO2 ratio <200. The use of ECMO for bilateral pulmonary edema on chest X-ray was classified grade 3. For assessments performed within 72 hours, a more severe threshold was incorporated.

Acute Cellular Rejection (ACR)

ACR is defined by the ISHLT working formulation as the presence of perivascular and interstitial mononuclear inflammatory infiltrates on transbronchial lung biopsy. Rejection is graded on a scale from A0 (no rejection) to A4 (severe rejection) based on the extent and character of the cellular infiltrate[13].

Antibody-Mediated Rejection (AMR)

AMR in lung transplantation is characterized by the presence of DSA along with histopathologic evidence of capillaritis and complement deposition in lung tissue, combined with clinical graft dysfunction. Transbronchial biopsies were performed for routine surveillance at 1, 3, 6, 9, and 12 months post-transplant, as well as at any time clinical suspicion for rejection arose. The diagnosis of AMR requires an integrated assessment of serologic, histologic, and clinical findings after excluding other potential causes of graft dysfunction[14].

Chronic Lung Allograft Dysfunction (CLAD)

CLAD is defined as a sustained (at least 3 months) decline in the forced expiratory volume in one second (FEV_1) of at least 20% from the post-transplant baseline, in the absence of other reversible causes. CLAD is further sub-classified into phenotypes such as bronchiolitis obliterans syndrome (BOS) and restrictive allograft syndrome (RAS) based on clinical, radiologic, and physiologic criteria [15].

Acute kidney injury (AKI)

AKI was defined using the Risk, Failure, Loss of kidney function, and End-stage kidney disease classification[16].

Respiratory Infections

Defined as the presence of clinical symptoms (fever, increased cough, purulent sputum, dyspnea) combined with radiographic evidence of new or progressive infiltrates, and confirmed by microbiological testing (e.g., sputum culture, bronchoalveolar lavage) [17].

Cytomegalovirus (CMV) Infections

Defined as the detection of CMV DNA via quantitative PCR with a cutoff viral load of ≥500 copies/mL within the first year post-transplant or positive antigenemia assay in the blood, along with clinical signs and symptoms (fever, malaise, organ-specific manifestations such as pneumonitis or hepatitis) [18].

Blood Culture–Positive Infections

Defined as the isolation of pathogenic bacteria or fungi from at least one set of blood cultures in a patient exhibiting clinical signs of systemic infection (e.g., fever, hypotension, leukocytosis). This follows the Centers for Disease Control and Prevention (CDC) criteria for healthcare-associated bloodstream infections [19].

Positive Aspergillus Galactomannan Antigen

Defined as an index value at or above the manufacturer's threshold (typically ≥0.5) in serum or bronchoalveolar lavage fluid, which is indicative of invasive aspergillosis. This definition is based on the European Organization for Research and Treatment of Cancer/Mycoses Study Group (EORTC/MSG) criteria for invasive fungal infections [20].

1. Fan E, Del Sorbo L, Goligher EC, et al. An official American Thoracic Society/European Society of intensive care medicine/society of critical care medicine clinical practice guideline: Mechanical ventilation in adult patients with acute respiratory distress syndrome. *Am J Respir Crit Care Med*. 2017;195(9). doi:10.1164/rccm.201703-0548ST

2. Badulak J, Antonini MV, Stead CM, et al. Extracorporeal Membrane Oxygenation for COVID-19: Updated 2021 Guidelines from the Extracorporeal Life Support Organization. *ASAIO Journal*. 2021;67(5). doi:10.1097/MAT.0000000000001422

3. Tomasko J, Prasad SM, Dell DO, DeCamp MM, Bharat A. Therapeutic anticoagulation–free

extracorporeal membrane oxygenation as a bridge to lung transplantation. *Journal of Heart and Lung Transplantation*. 2016;35(7). doi:10.1016/j.healun.2016.04.005

4. Kurihara C, Walter JM, Karim A, et al. Feasibility of Venovenous Extracorporeal Membrane Oxygenation Without Systemic Anticoagulation. In: *Annals of Thoracic Surgery*. Vol 110. ; 2020. doi:10.1016/j.athoracsur.2020.02.011

5. Yu WS, Paik HC, Haam SJ, et al. Transition to routine use of venoarterial extracorporeal oxygenation during lung transplantation could improve early outcomes. *J Thorac Dis*. 2016;8(7). doi:10.21037/JTD.2016.06.18

6. Yagi Y, Manerikar A, Bharat A, Kurihara C. Central VA-ECMO Support without Full Anticoagulation is Feasible During Lung Transplant. *The Journal of Heart and Lung Transplantation*. 2022;41(4). doi:10.1016/j.healun.2022.01.1490

7. Mitzman B. Commentary: Taking matters into your own hands: Venoarterial extracorporeal membrane oxygenation for controlled reperfusion during lung transplantation. *Journal of Thoracic and Cardiovascular Surgery*. 2020;160(1). doi:10.1016/j.jtcvs.2019.11.037

8. Halpern SE, Wright MC, Madsen G, et al. Textbook outcome in lung transplantation: Planned venoarterial extracorporeal membrane oxygenation versus off-pump support for patients without pulmonary hypertension. *Journal of Heart and Lung Transplantation*. 2022;41(11). doi:10.1016/j.healun.2022.07.015

9. Chan EG, Hyzny EJ, Furukawa M, et al. Intraoperative Support for Primary Bilateral Lung Transplantation: A Propensity-Matched Analysis. *Annals of Thoracic Surgery*. 2023;115(3). doi:10.1016/j.athoracsur.2022.06.014

10. Andreasson A, Hoetzenecker K. Commentary: Why a routine venoarterial extracorporeal membrane oxygenation support strategy is a good idea in lung transplantation. *Journal of Thoracic and Cardiovascular Surgery*. 2022;164(5). doi:10.1016/j.jtcvs.2021.12.008

11. Toyoda T, Kurihara C, Kaiho T, et al. Predictors of Cytomegalovirus Recurrence Following Cessation of Posttransplant Prophylaxis. *Journal of Surgical Research*. 2024;299:129-136. doi:10.1016/j.jss.2024.04.012

12. Snell GI, Yusen RD, Weill D, et al. Report of the ISHLT Working Group on Primary Lung Graft Dysfunction, part I: Definition and grading—A 2016 Consensus Group statement of the International Society for Heart and Lung Transplantation. *Journal of Heart and Lung Transplantation*. 2017;36(10). doi:10.1016/j.healun.2017.07.021

13. Stewart S, Fishbein MC, Snell GI, et al. Revision of the 1996 Working Formulation for the Standardization of Nomenclature in the Diagnosis of Lung Rejection. *Journal of Heart and Lung Transplantation*. 2007;26(12). doi:10.1016/j.healun.2007.10.017

14. Levine DJ, Glanville AR, Aboyoun C, et al. Antibody-mediated rejection of the lung: A consensus report of the International Society for Heart and Lung Transplantation. *Journal of Heart and Lung Transplantation*. 2016;35(4). doi:10.1016/j.healun.2016.01.1223

15. Verleden GM, Glanville AR, Lease ED, et al. Chronic lung allograft dysfunction: Definition, diagnostic criteria, and approaches to treatment—A consensus report from the Pulmonary Council of the ISHLT. *Journal of Heart and Lung Transplantation*. 2019;38(5). doi:10.1016/j.healun.2019.03.009

16. Bellomo R, Ronco C, Kellum JA, Mehta RL, Palevsky P. Acute renal failure - definition, outcome measures, animal models, fluid therapy and information technology needs: the Second International Consensus Conference of the Acute Dialysis Quality Initiative (ADQI) Group. In: *Critical Care (London,*

England). Vol 8. ; 2004. doi:10.1186/cc2872

17. Kalil AC, Metersky ML, Klompas M, et al. Management of Adults With Hospital-acquired and Ventilator-associated Pneumonia: 2016 Clinical Practice Guidelines by the Infectious Diseases Society of America and the American Thoracic Society. *Clinical Infectious Diseases*. 2016;63(5). doi:10.1093/cid/ciw353

18. Kotton CN, Kumar D, Caliendo AM, et al. The Third International Consensus Guidelines on the Management of Cytomegalovirus in Solid-organ Transplantation. *Transplantation*. 2018;102(6). doi:10.1097/TP.0000000000002191

19. Centers for Disease Control and Prevention. National Healthcare Safety Network (NHSN) Patient Safety Component Manual. *National Healthcare Safety Network (NHSN) Patient Safety Component Manual*. 2024;(January).

20. De Pauw B, Walsh TJ, Donnelly JP, et al. Revised definitions of invasive fungal disease from the European Organization for Research and Treatment of Cancer/Invasive Fungal Infections Cooperative Group and the National Institute of Allergy and Infectious Diseases Mycoses Study Group (EORTC/MSG) Consensus Group. *Clinical Infectious Diseases*. 2008;46(12). doi:10.1086/588660
